# *Stenotrophomonas maltophilia* bloodstream infection in patients with hematologic malignancies: a retrospective study and in vitro activities of antimicrobial combinations

**DOI:** 10.1186/s12879-015-0801-7

**Published:** 2015-02-18

**Authors:** Sung-Yeon Cho, Dong-Gun Lee, Su-Mi Choi, Chulmin Park, Hye-Sun Chun, Yeon-Joon Park, Jae-Ki Choi, Hyo-Jin Lee, Sun Hee Park, Jung-Hyun Choi, Jin-Hong Yoo

**Affiliations:** Division of Infectious Diseases, Department of Internal Medicine, College of Medicine, The Catholic University of Korea, St. Mary’s Hospital, 222, Banpo-daero, Seocho-gu, Seoul Republic of Korea; Vaccine Bio Research Institute, College of Medicine, The Catholic University of Korea, St. Mary’s Hospital, 222, Banpo-daero, Seocho-gu, Seoul Republic of Korea; The Catholic Blood and Marrow Transplantation Center, College of Medicine, The Catholic University of Korea, St. Mary’s Hospital, 222, Banpo-daero, Seocho-gu, Seoul Republic of Korea; Department of Laboratory Medicine, College of Medicine, The Catholic University of Korea, St. Mary’s Hospital, 222, Banpo-daero, Seocho-gu, Seoul Republic of Korea

**Keywords:** Bacteremia, Drug combinations, Hematologic diseases, Mortality, *Stenotrophomonas maltophilia*

## Abstract

**Background:**

*Stenotrophomonas maltophilia* causes serious infections in immunocompromised hosts. Here, we analyzed the clinical characteristics of *S. maltophilia* bloodstream infection (BSI) in patients with hematologic malignancies and evaluated in vitro synergistic effects of antimicrobial combinations.

**Methods:**

We retrospectively reviewed all consecutive episodes of *S. maltophilia* BSIs in adult hematologic patients from June 2009 to May 2014, with in vitro susceptibility and synergy tests using high-throughput bioluminescence assay performed for available clinical isolates.

**Results:**

Among 11,004 admissions during 5-year period, 31 cases were identified as *S. maltophilia* BSIs. The incidence rate of *S. maltophilia* BSI was 0.134 cases/1,000 patient-days. Overall and attributable mortality of *S. maltophilia* BSI was 64.5% and 38.7%, respectively. Severe neutropenia (adjusted hazard ratio [HR] 5.24, *p* =0.013), shock at the onset of BSI (adjusted HR 6.05, *p* <0.001), and pneumonia (adjusted HR 3.15, *p* =0.017) were independent risk factors for mortality. In vitro susceptibilities to ceftazidime, levofloxacin, ticarcillin-clavulanic acid (TIM) and trimethoprim-sulfamethoxazole (SXT) were 11.1%, 44.0%, 40.7%, and 88.9%, respectively. MIC_50_/MIC_90_ for moxifloxacin and tigecycline were 1/4 mg/L and 4/8 mg/L. The 50% and 90% fractional inhibitory concentrations (FIC^50^/FIC^90^) of clinical isolates against a combination of SXT and TIM were 0.500/0.750. For SXT plus levofloxacin or moxifloxacin, FIC^50^/FIC^90^ were 0.625/1.000 and 0.625/0.625, respectively.

**Conclusion:**

*S. maltophilia* BSIs show high mortality, which is related to severe neutropenia, shock, and *S. maltophilia* pneumonia. Based upon drug susceptibility testing, the primary treatment of choice for *S. maltophilia* BSIs should be SXT in hematologic patients, rather than quinolones, with combination therapies including SXT serving as a feasible treatment option.

## Background

*Stenotrophomonas maltophilia* is an emerging nosocomial pathogen in immunocompromised patients [1-3]. Although *S. maltophilia* exhibits a limited pathogenicity in immunocompetent hosts, it has been shown to cause fatal infections in patients with hematologic malignancies. The overall mortality of *S. maltophilia* bloodstream infections (BSIs) ranges from 21 to 50%, with the mortality associated with neutropenia [4-6]. Failure to administration of early susceptible antibiotics for *S. maltophilia* BSI can have clinical implications, as *S. maltophilia* is naturally resistant to many antimicrobial agents including carbapenem.

Trimethoprim-sulfamethoxazole (SXT) is the antimicrobial agent of choice for the treatment of *S. maltophilia* infections [7-9]. Levofloxacin is also a viable treatment option in cases where drug susceptibilities are known [10]. However, SXT is known to cause adverse events related to bone marrow suppression, which might delay recovery from neutropenia in patients with hematologic malignancies. Fluoroquinolone is commonly used as prophylaxis during stem cell transplantation (SCT) or chemotherapy. As recent guidelines and experts have suggested that there are concerns about potential resistance to fluoroquinolone-based prophylaxis, this prophylactic strategy can lead to a limited effectiveness of levofloxacin in *S. maltophilia* infections [7,11-13]. Data regarding the clinical characteristics and the treatment outcomes of *S. maltophilia* BSIs in hematologic patients who received quinolone prophylaxis remain insufficient.

As *S. maltophilia* BSIs are associated with a high mortality rate, and increased resistance to monotherapy, many groups have suggested the need for combination antimicrobial therapies [7,14,15]. However, the effectiveness of combination therapy for *S. maltophilia* has not yet been established. Here, we investigated the clinical characteristics and outcomes related to *S. maltophilia* BSIs in patients with hematologic malignancies. Clinical isolates from these patients were then evaluated for in vitro susceptibilities with synergistic effects of several antimicrobial combinations to identify potential therapeutic regimens that may improve clinical outcomes.

## Methods

### Study design and hospital setting

We retrospectively reviewed medical records of all consecutive episodes of *S. maltophilia* BSIs in adult patients with hematologic malignancies from June 2009 to May 2014 at the Catholic Blood and Marrow Transplantation Center of Seoul St. Mary’s Hospital.

### Clinical data collection

Eligible patients included those with hematologic malignancies older than 19 years of age, with documented blood cultures positive for *S. maltophilia*. Clinical data obtained for each patient included age, sex, underlying diseases, severity and duration of neutropenia, length of hospital stay, simplified acute physiology score II (SAPS II) at the onset of BSI, the presence of central venous catheters, organisms isolated from blood and the antimicrobial susceptibility, administered antibiotics, and survival status at 30 days after the onset of BSI. The Institutional Review Board of Seoul St. Mary’s Hospital approved the research protocol and waived the requirement for informed consent (KC13SISI0163).

### *S. maltophilia* 16S rRNA gene analysis & pulsed-field gel electrophoresis

Available clinical isolates underwent phylogenetic group determination and pulsed-field gel electrophoresis (PFGE). Clinical isolates were screened using a specific 16S rRNA gene polymerase chain reaction (PCR) assay, and sequenced to confirm taxonomic identities. PCR was performed using primers SM1f (5′-GTTGGGAAAGAAATCCAGC-3′) and SM4 (5′-TTAAGCTTGCCACGAACAG-3′) as described previously [16,17]. Sequence analysis of PCR products was conducted with MEGA version 3.1 using the maximum likelihood method. AB695350 (*S. maltophilia* strain 4APB) was used as a control [18]. *S. maltohpilia* clinical isolates were typed using PFGE with *Xba* I digestion as described previously [19]. PFGE was performed with a CHEF-DR III apparatus (Bio-Rad Korea, Seoul, Korea) using 5 to 35 s of linear ramping at 6 V/cm for 20 h at 14°C. Digital images were analyzed with Fingerprinting II Informatix software (Bio-Rad, Hercules, CA, USA) using the Dice coefficient and UPGMA with a 1% tolerance and 0.5% optimizing setting value. The results were interpreted using the criteria of Tenover et al. [20].

### Antimicrobial susceptibilities and fractional inhibitory concentrations using a luciferase-based assay

An in vitro susceptibility test was performed for seven antimicrobial agents (ceftazidime, ciprofloxacin, levofloxacin, moxifloxacin, ticarcillin-clavulanic acid [TIM], tigecycline, and SXT) using the broth microdilution method according to 2013 Clinical and Laboratory Standards Institute guidelines [21]. Quality controls were assessed by using *Escherichia coli* ATCC 25922 and *Pseudomonas aeruginosa* ATCC 27853. TIM was obtained from Biovim Korea Vine & Company (Seoul, Korea). Tigecycline was obtained from Pfizer Inc. (New York, NY, USA) via a compound transfer program. Other antibiotics were obtained from Sigma-Aldrich (St. Louis, MO, USA). All susceptibility testing was performed using cation-adjusted Mueller-Hinton broth (BD, Spark, MD, USA). To identify synergistic effects between SXT and other antibiotics (levofloxacin, moxifloxacin, or TIM), a checkerboard assay was performed using 96-well U-bottom microplates. Due to a previous report that broth microdilution endpoint for SXT are difficult to read because of trailing and bacteriostatic activity of *S. maltophilia*, a luciferase-based bacterial cell viability assay was used [22,23]. In this study, a BacTiter-Glo™ microbial cell viability kit (Promega Corp., Madison, WI, USA) was used to determine the number of viable bacterial cells in culture, based on quantification of adenosine triphosphates (ATPs). Graded concentrations of antibiotics were mixed to assess synergy test. Each well was inoculated with 5 × 10^4^ CFU of each isolate in cation-adjusted Mueller-Hinton broth. The plates were then incubated for 24 h at 35°C in ambient air. All assays were performed in triplicate. After 24 h of incubation, a volume of BacTiter-Glo™ reagent equal to the volume of the cell culture medium was added to 100 μL of microbial broth culture in an opaque-walled multi-well plate, according to the manufacturer’s instructions. Relative luminescence units (RLU) were measured using a SpectraMax L luminescence microplate reader (Molecular Devices, Sunnyvale, CA, USA). The percentage of RLUs compared to the antibiotic-free controls (%RLU) was calculated, with minimal inhibitory concentrations (MICs) defined as <10%RLU, corresponding to an inhibitory concentration of 90% (IC_90_). Total fractional inhibitory concentrations (FIC) were calculated according to the formula: ΣFIC = FIC of agent A + FIC of agent B, where FIC of agent A or B = MIC of agent A or B in combination/MIC of agent A or B alone. ΣFIC values ≤0.5 indicate synergy, ΣFIC values of >0.5 and ≤4 indicate indifference, and ΣFIC values >4 indicate antagonism [24].

### Definitions

*S. maltophilia* BSIs were defined as at least one *S. maltophilia*-positive blood culture in association with clinical signs or symptoms indicative of infection [6]. Polymicrobial BSIs were defined as the presence of an organism other than *S. maltophilia* in the same blood culture. The source of bacteremia was determined clinically on the basis of the presence of an active site of infection as determined by chart review or isolation of the organism from other clinical specimens coincident with the episode of bacteremia [4]. Neutropenia was defined as an absolute neutrophil count (ANC) <500/mm^3^, or <1000/mm^3^ with predicted falls to <500/mm^3^ within 2–3 days. Severe neutropenia was defined as an ANC <100/mm^3^ [11,12]. The length of hospitalization before BSI was defined as the number of days from hospital admission to the development of BSI. Previous antibiotic use was defined as the administration of antibiotics for more than 24 hours within 30 days before the onset of the *S. maltophilia* BSI [25]. Mortality was considered attributable to the *S. maltophilia* BSI in any of the following cases: (1) blood cultures positive for *S. maltophilia* at the time of death; (2) death before the resolution of signs and symptoms related to *S. maltophilia* BSI; (3) death within 7 days of the onset of *S. maltophilia* BSI and with no other identifiable cause [26]. Crude mortality was defined as mortality that occurred within a month following a BSI episode [27].

### Statistical analysis

Differences in continuous variables between the survivors and non-survivors were analyzed using the Mann–Whitney *U*-test. Fisher’s exact test was used to compare categorical data. We used Cox’s proportional hazard model with forward stepwise selection to identify independent risk factors for death. Kaplan-Meier survival curves were used to analyze mortality trends. *P* value <0.05 was considered statistically significant. All data were analyzed using SPSS ver. 18.0 (SPSS Korea, Seoul, Korea).

## Results

### Clinical characteristics of *S. maltophilia* bloodstream infection

Among 11,004 of admission episodes, a total of 31 patients were treated for *S. maltophilia* BSI. The incidence rate of *S. maltophilia* BSI was 0.134 cases per 1,000 patient-days during the entire study period. All patients had received broad-spectrum antibiotics such as fluoroquinolone as prophylaxis, anti-Pseudomonal cephalosporin plus aminoglycoside, or carbapenem as empirical or targeted therapy due to neutropenic fever within 30 days before the onset of the BSIs. The most commonly identified source of BSI was pneumonia (41.9%), followed by primary BSI (22.6%), catheter-related BSI (19.4%), skin and soft tissue infection (12.9%), and intra-abdominal infection (3.2%). In catheter-related *S. maltophilia* BSIs, Hickman catheters (n = 5) or chemoport (n = 1) were removed from the patient. In addition, about one-third of the patients (35.5%) had polymicrobial BSIs with nosocomial pathogens such as methicillin-resistant *Staphylococcus aureus*, methicillin-resistant coagulase-negative *Staphylococcus*, *P. aeruginosa* or vancomycin-resistant *Enterococcus*. Although antibiotic regimens were modified to appropriate targeted therapies in 26 of 31 patients (83.9%), the overall and attributable mortality of *S. maltophilia* BSIs was 64.5% and 38.7%, respectively.

Clinical characteristics of *S. maltophilia* BSIs were compared according to overall survival status (Table 1). There were no differences in age, sex, underlying hematologic diseases, SAPS II, presence of polymicrobial BSI, or shock between the two groups. Neutropenia at the onset of BSI (43% vs. 92%, *p* =0.038) was significantly associated with death, in terms of both neutropenia itself and the duration of neutropenia (median 3 d vs. 40 d, *p* =0.016). As a source of BSI, pneumonia (0% vs. 65.0%, *p* =0.001) was more common in non-survivors, while catheter-related infections (54.5% vs. 0%, *p* =0.001) were more common in survivors. In patients with combined *S. maltophilia* pneumonia, 64.5% (8 of 13 patients) received mechanical ventilation, while 11.1% (2 of 18 patients) with other sources of BSI received mechanical ventilation.

**Table 1:** **Clinical characteristics of patients with** ***Stenotrophomonas maltophilia*** **bloodstream infections according to the overall survival status**

**Variable**	**Survival (n = 11)**	**Death (n = 20)**	***P*** **value**
Age	49 (22–65)	48 (18–78)	0.416
Male	6 (54.5%)	12 (60.0%)	0.999
Underlying disease			
Myeloid malignancies	6 (54.5%)	15 (75.0%)	0.453
Lymphoid malignancies	3 (27.3%)	3 (15%)	
Others	2 (18.2%)	2 (10.0%)	
Treatment			
Chemotherapy	8 (72.7%)	9 (45.0%)	0.089
Stem cell transplantation	3 (27.3%)	4 (20.0%)	
Palliative care	0 (0.0%)	7 (35.0%)	
Hospital stay, days	26 (0–48)	23.5 (0–120)	0.119
Duration of neutropenia^a^, days	3 (0–36)	40 (1–135)	0.016
Neutropenia at the onset of BSI (<500/mm^3^)	7 (63.6%)	19 (95.0%)	0.042
Severe neutropenia at the onset of BSI (<100/mm^3^)	6 (54.5%)	17 (85.0%)	0.095
SAPS II	30 (26–49)	38.5 (23–70)	0.030
Source of infection			
Pneumonia	0 (0.0%)	13 (65.0%)	0.001
Catheter	6 (54.5%)	0 (0.0%)	0.001
Primary	3 (27.3%)	4 (20.0%)	0.999
SSTI	1 (9.1%)	3 (15.0%)	0.999
Abdomen	1 (9.1%)	0 (0.0%)	0.355
Polymicrobial BSI	3 (27.3%)	7 (35.0%)	0.999
Shock	2 (18.2%)	12 (60.0%)	0.057

Data are presented as n (%) or median (range).

*Abbreviations:* BSI, bloodstream infection; SAPS II, Simplified Acute Physiology Score II; SSTI, skin and soft tissue infection.

^a^missing data (n = 11).

**Figure 1:**
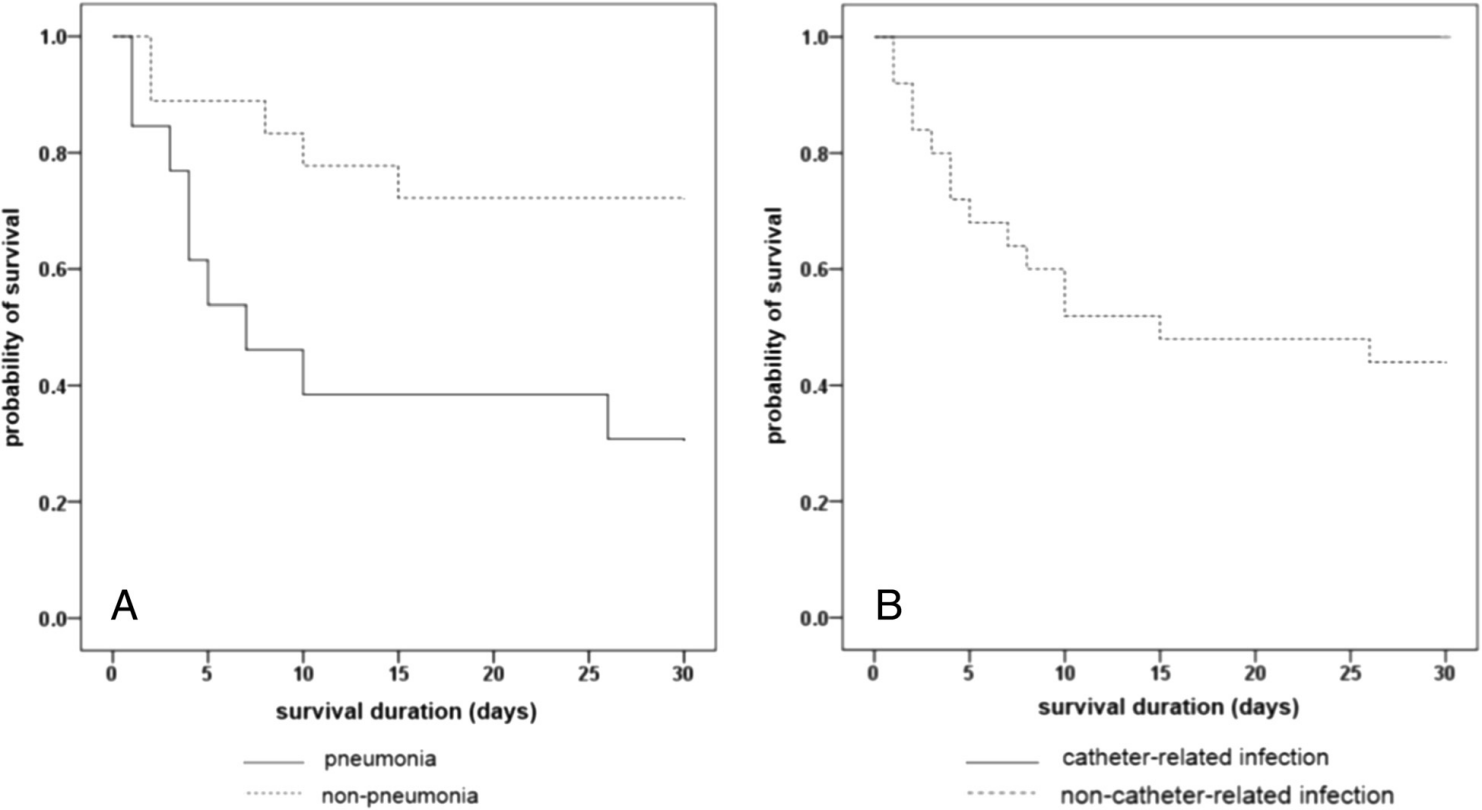
**Kaplan-Meier curves for survival during episodes of** ***Stenotrophomonas maltophilia*** **bloodstream infections.** Kaplan–Meier curves for survival stratified by pneumonia as a source of BSI **(A)**, and catheter-related BSI **(B)** (Log-rank test, *p* <0.001, and 0.003, respectively).

The results of Cox’s proportional hazard analysis of factors associated with overall mortality are shown in Table 2. Severe neutropenia at the onset of BSI (adjusted hazard ratio [HR] 5.24, 95% Confidence Interval [CI] 1.411-19.493; *p* =0.013) and shock (adjusted HR 3.15, 95% CI 1.231-8.032; *p* =0.017) at the onset of BSI, and pneumonia as a source of BSI (adjusted HR 6.05, 95% CI 2.247-16.291; *p* <0.001) were associated with an increase in mortality. Kaplan-Meier survival curves stratified by source of BSI were shown in Figure 1.

**Table 2:** **Factors associated with mortality in patients with** ***Stenotrophomonas maltophilia*** **bloodstream infections**

	**Univariate analysis**		**Multivariate analysis**	
**Variable**	**HR (95% CI)**	***P*** **value**	**Adjusted HR (95% CI)**	***P*** **value**
Neutropenia at the onset of BSI (<500/mm^3^)	5.87 (0.784-44.037)	0.085		
Severe neutropenia at the onset of BSI (<100/mm^3^)	3.47 (1.008-11.954)	0.048	5.24 (1.411-19.493)	0.013
Duration of neutropenia >21 days	3.29 (0.978-11.072)	0.054		
Hospital stay >30 days	1.44 (0.586-3.531)	0.428		
SAPS II >40	3.14 (1.265-7.783)	0.003		
Source of infection				
Pneumonia	4.27 (1.687-10.816)	0.002	6.05 (2.247-16.291)	<0.001
Catheter	0.03 (0.000-1.817)	0.093		
Polymicrobial BSI	1.43 (0.570-3.603)	0.444		
Shock	3.09 (1.245-7.666)	0.015	3.15 (1.231-8.032)	0.017

*Abbreviations:* BSI, bloodstream infection; CI, confidence interval; HR, hazard ratio; SAPS II, Simplified Acute Physiology Score II; SSTI, skin and soft tissue infection

### Pulsed-field gel electrophoresis and phylogenetic group determinations

PFGE typing of available 27 *S. maltophilia* clinical isolates demonstrated 20 distinguishable banding patterns without evidence of the clonality. These 20 strains exhibited >99.9% 16S rRNA gene sequence similarity to the *S. maltophilia* type strain AB695350.

### In vitro susceptibility testing and antimicrobial combinations

Broth microdilution testing of clinical isolates revealed 88.9% susceptibility to SXT and 44.4% to levofloxacin. MIC_50_ and MIC_90_ values for moxifloxacin and tigecycline showed 1 and 4 mg/L, and 4 and 8 mg/L, respectively (Table 3). Of the 27 clinical isolates, 15 were chosen for additional synergy testing based upon their MIC values. The selected isolates were representatives of the similar MIC patterns to each antmicrobial agent. Comparison of FIC indices is shown in Table 4. Synergy between SXT and TIM was found in 9 of 15 strains (60%) tested, of which the FIC for 50 and 90% of the isolates (FIC^50^/FIC^90^) was 0.500/0.750, with a range of 0.254 to 1.500. In the case of SXT plus levofloxacin or moxifloxacin, FICs ranged from 0.500 to 1.000, and 0.313 to 0.750, respectively. In all of the antimicrobial combinations tested, based on the SXT, the FIC^90^ values were under 1.000 without antagonism.

**Table 3:** **In vitro activity of antimicrobial agents against** ***Stenotrophomonas maltophilia*** **isolates**

**Antimicrobial agent**	**Susceptible isolates (%)**	**MIC range (mg/L)**	**MIC**_**50**_ **(mg/L)**	**MIC**_**90**_ **(mg/L)**
CAZ	11.11	4 ~ ≥128	32	≥128
CIP	0	4 ~ ≥ 64	32	64
LVX	44.44	0.5 ~ 16	4	8
MXF	-	0.125 ~ 8	1	4
SXT	88.89	0.25/4.75 ~ 4/76	0.25/4.75	4/76
TGC	-	1 ~ 8	4	8
TIM	40.74	0.5/2 ~ ≥512/2	32/2	128/2

*Abbreviations:* CAZ, ceftazidime; CIP, ciprofloxacin; LVX, levofloxacin; MXF, moxifloxacin; SXT, trimethoprim-sulfamethoxazole; TGC, tigecycline; TIM, ticarcillin-clavulanic acid

**Table 4:** **Comparison of fractional inhibitory concentration ranges for each antimicrobial combination**

**Antimicrobial combination**	Σ**FIC** ^**a**^			**Synergy (%)**	**Indifference (%)**	**Antagonism (%)**
	**50%**	**90%**	**Range**			
SXT plus TIM	0.500	0.750	0.254 to 1.500	60	40	0
SXT plus LVX	0.625	1.000	0.500 to 1.000	40	60	0
SXT plus MXF	0.625	0.625	0.313 to 0.750	40	60	0

*Abbreviations*: FIC, fractional inhibitory concentration; LVX, levofloxacin; MXF, moxifloxacin; SXT, trimethoprim-sulfamethoxazole; TIM, ticarcillin-clavulanic acid.

^a^50% and 90%, ΣFIC for 50% and 90% of the isolates, respectively.

## Discussion

In this study, we examined the clinical characteristics and treatment outcomes in hematologic patients with *S. maltophilia* BSIs, as well as the effectiveness of in vitro antimicrobial combinations against *S. maltophilia* clinical isolates. *S. maltophilia* BSIs still showed high mortality, with significant correlations seen for severe neutropenia, shock, and concomitant *S. maltophilia* pneumonia. Furthermore, we discovered that in vitro synergy tests revealed favorable FIC^50^ and FIC^90^ values against *S. maltophilia* clinical isolates obtained from hematologic patients.

Our data demonstrated that the previous exposure to broad spectrum antibiotics was preceded in all of the *S. maltophilia* BSIs. Polymicrobial infections were observed in over one third of patient. Poor prognosis in patients with combined *S. maltophilia* pneumonia might be related to higher rates of mechanical ventilation, when compared to patients with other sources of BSI. In contrast, patients with catheter related BSIs exhibited 100% survival in this study. Favorable outcomes in these cases are likely to be associated with the removal of the catheter. A previous retrospective study found that catheter-related *S. maltophilia* BSIs were cured after removal of the catheter, even without appropriate antibiotics therapy, while a different study observed a significant correlation between mortality and retained catheters [25,28]. However, these studies were limited by the small number of patients examined, and failed to evaluate other salvage management such as antibiotics lock therapy in patients using long-term catheters.

In a worldwide study of the antimicrobial susceptibilities of 1,586 clinical *S. maltophilia* isolates, SXT and tigecycline showed susceptibilities of 96.0% and 95.5%, respectively, followed by levofloxacin with a susceptibility of 83.4% [29]. A recent study reported no difference in 30-day mortality between SXT and levofloxacin treatment for *S. maltophilia* BSIs [10]. However, in vitro susceptibility results from our study also revealed characteristics indicative of patient therapies. Decreased susceptibility to levofloxacin was associated with prophylactic fluoroquinolone use during the chemotherapy or SCT for hematologic malignancies. Taken together, levofloxacin may be an appropriate alternative treatment for *S. maltophilia* infections though careful patient selection based on the history of previous fluoroquinolone exposure will be necessary. For SXT, the susceptibility rate was 88.9% using the broth microdilution method, compared to 93.9% using an automated system (Vitek-2, bioMérieux, Hazelwood, MO, USA). This difference is likely the result of the inoculum effect and trailing. The MIC_50/90_ for moxifloxacin and tigecycline also showed higher values, and thus needs further evaluation for the clinical use in this group of patients.

In vitro synergy was screened for SXT in combination with other antimicrobial agents. About 90% of *S. maltophilia* isolates were susceptible to SXT, though several reports of emerging resistance to SXT have been found [29]. We included TIM as a representative of the beta-lactam antibiotics, as a result of our in vitro susceptibility tests. Fluoroquinolone was selected for synergy test due to its widespread clinical use. Another reason to choose quinolone as a combination antibiotics was the possible activity against biofilm formation in device-related infection or in cystic fibrosis patients [30,31]. Further studies are needed to identify the biofilm activity of quinolone in hematologic patients. Our study demonstrated that SXT plus TIM exhibited the highest rates of synergy among the antibiotic combinations tested. Both SXT plus levofloxacin and SXT plus moxifloxacin revealed FIC below 1.000 against all of the clinical isolates tested. Combination therapy may, therefore, represent a viable option for *S. maltophilia* BSIs. Further studies with larger number of patients will be needed to assess whether the combination therapy has clinical impact in improving outcomes of *S. maltophilia* BSI in hematologic patients.

There are several strengths of this study. First, only BSI cases were included. In non-bacteremic *S. maltophilia* infection, it is difficult to distinguish the colonization from infection, which might influence the outcome analysis. Second, we calculated the incidence rate of *S. maltophilia* BSI in hematologic patients. Third, we used a high-throughput bioluminescence assay to assess the viability in living organisms, and a luciferase-based assay for determining the number of viable cells in culture [32]. The application of the luciferase-based assay for the measurement of FIC indices was used to overcome difficulties in reading due to trailing endpoint by converting MIC to %RLU, equivalent to IC_90_ values.

## Conclusion

In conclusion, *S. maltophilia* BSIs shows high mortality in patients with hematologic malignancies. Neutropenia, shock, and combined *S. maltophilia* pneumonia are associated with mortality. Based upon drug susceptibility testing, the primary treatment of choice in hematologic patients should be SXT, with combination therapies including SXT serving as a feasible treatment option for *S. maltophilia* BSIs.
